# ICT diffusion and financial development: Comparing high, middle, and low-income countries

**DOI:** 10.1371/journal.pone.0295183

**Published:** 2024-05-02

**Authors:** Ying Li, XiaoGuang Li, Haseeb Ahmad

**Affiliations:** 1 The Tourism College of Changchun University, Changchun City, Jilin Province, China; 2 Department of Computer Science, National Textile University, Faisalabad, Pakistan; Lahore School of Economics, PAKISTAN

## Abstract

Given the importance of ICT diffusion in the development of the financial sector, this analysis is an effort to analyze the transmission channels between the two in high-income and middle and low-income economies over 2001–2019. We have used three variables, including the ICT index, individuals using the internet, and mobile subscribers, to represent ICT and three indices, including the financial development index, financial institution index, and financial market index, to make our results reliable and robust. We utilized a GMM method for conducting the empirical analysis. Generally, our results imply that ICT diffusion positively impacts financial development in high-income economies and negatively impacts middle and low-income economies. Our findings suggest that middle- and low-income-economy policymakers should follow the footprint of the high-income economies and increase the role of ICT in the financial sector for its development.

## Introduction

An unprecedented development in ICT is influencing all spheres of human life. Particularly, it has fundamentally transformed the ways information is managed and processed by Banks and financial institutions. The worldwide financial sector’s ICT expenses were more than US$197 billion in 2014, demonstrating a greater share of ICT investment than any other sector from 1995 [1]. Meanwhile, financial development is also transforming its speed and shape along with ongoing ICT diffusion. ICT advancements in the corporate sector per se provide unique trade opportunities for operational and prospective firms by offering innovative ways of value creation and devising novel ways of product and service delivery [2].

Theoretical mechanisms which explain ICT diffusion and financial development can be categorized as follows. First, lower cost: Cloud computation can offer the same payment services at a lower cost, which needs advanced computation infrastructure and capacities [3]. Second, data applications: the greater use of data applications in the financial industry helps to forecast customers’ preferences, demands, and market trends. The better information helps to manage and deliver suitable financial products and services matching the best time and the best price [4]. Third, efficient supervision: ICT diffusion in the banking sector can support internal risk supervision and anticipate loan default chances. In this way, the objectives of consumer protection and regulations are easily achieved [5]. Besides, investing in the blockchain industry helps to maintain a permanent record of digital data and enhances mutual trust by reducing the chances of data manipulation [6, 7]. Fourth, favorable diffusion, ICT favorably influences the financial sector by augmenting the performance of financial institutions overcoming the issues of asymmetric information in financial dealings.

Recently, ICT diffusion has created a new challenge for the conventional financial industry by spurring the increase in financial technology (FinTech). The emerging Fintech can stifle the conventional financial sector by reforming the present structure of the financial systems and boosting competition between alternative financial structures and stakeholders [8]. The emerging FinTech corporations offer the same products and services as do banks adapting a diverse and unbundled mode [9]. Particularly, these corporations have a competitive edge owing to the fact they are using advanced technology. Thus, ICT applications have penetrated rapidly in the financial industry, however, the influence of ICT diffusion on financial development remains unclear [6].

Since ICT development is making financial markets more efficient, capital costs are declining. The academic literature highlights the ICT and financial development nexus. In this respect, Allen et al. [10] and Domowitz [11] argued that electronic finance technology overcomes information asymmetries between customers and producers and costs associated with data handling. Purcell and Toland [12] suggested that ICT plays a greater role in managing credit information data records and delivering debtors and institutional references. Shamim [13] and Majeed and Ayub [14] exhibited that ICT can support economic growth by decreasing operational and informational expenses. In the study conducted by Pradhan [15], the empirical findings from their research validate the presence of causal relationships between ICT and financial development in the economies of Asia. Majeed and Malik [16] argued that ICT implementation in the public sector plays a conducive role in influencing the financial sector by alleviating market distortions, transaction costs, information costs, and improving market information. Furthermore, ICT helps to optimize resource allocation in the financial industry by directing resource allocation from surplus to deficit sectors by disseminating information. Moreover, they showed in cross-sectional data of 147 economies that ICT in the public sector moderates the power of financial development in explaining cross-country growth variations.

One strand of the literature focuses on inclusive financial development or financial access. For example, using GMM and quantile regression and the data for 162 Banks in African economies, Asongu et al. [17] demonstrated that ICT penetration mitigates the exploiting impacts of market power on loan price and quantity. Besides, ICT also increases financial access by altering the influence of ICT on market power. Contrary to this, Peruta [18] found out that mobile money use has an insignificant impact on financial access. Using 2013 company data in Africa, Gosavi [19] showed that mobile money supports firms in getting loans and increases firms’ productivity. Another strand of the data focuses on formal and informal financial development. In this regard, Asongu [20], using the data from 53 African economies and OLS regression, showed that an increase in mobile phones adversely influences formal financial development and favorably affects formal financial development. In another study, Asongu and Acha-Anyi [21] explored ICT and financial development nexus for 53 African economies considering different levels of financial development and employing the quantile regressions approach. Their results suggest that the positive complement impact of ICT and financial formalization enhances financial development while the negative complement impact of ICT and financial informalization lowers financial development.

It is argued that internet use can negatively influence financial development owing to the fact that economic agents can directly conduct transactions. On the other hand, the mobile phone is used as a tool for communication, and it is less likely to conduct transactions on the phone. In this situation intermediaries are required to complete the financial transaction [22, 23]. Likewise, Ilyina and Samaniego [24] argued that ICT diffusion in financial systems increases volatility and sensitivity which in turn drives profits of transaction and financial instability. Thus, there is a controversy on ICT and financial development nexus. Chien et al. [1] investigated the nonlinear effects of ICT on financial development using the panel data of 81 economies from 1990 to 2015 and employing the GMM approach. Their findings provide mixed evidence on ICT and financial development association depending upon the ICT measure. Particularly, they showed that a lower level of ICT improves financial development while a higher level of ICT deteriorates financial development.

Nguyen et al. [22] provide global evidence on ICT and financial development nexus using the data from 1998 to 2017 and the GMM approach. They also revealed mixed evidence depending on the measures of ICT and financial development. Their findings show that internet use is negatively and significantly associated with different measures of financial development. On the other hand, mobile use is positively and significantly associated with all measures of financial development. Recently, Ejemeyovwi et al. [25] analyzed the influence of ICT and innovation on financial development using a sample of 54 Africans from 2000 to 2017. The results demonstrated that ICT-innovation shock positively influences financial development in the selected economies. Using the panel data of 34 African countries from 2005 to 2017, Mignamissi [26] demonstrated ICT divide is the main obstacle to the development of the financial sector in Africa. A detailed summary of the literature is also reported in Table 1. The current state of research in the field lacks comprehensive analysis and empirical evidence regarding the impact of ICT on financial development across various income groups. One critical research gap is the absence of large-scale studies that encompass a wide range of economies, including both high-income and middle and low-income countries.

**Table 1:** Summary of literature.

Author(s)	Region/Country	Time span	Methods	Independent variables	Outcomes
Edo et al. [27]	Nigeria and Kenya	2000–2016	DOLS& VECM	Internet users	Positive
Nguyen et al. [22]	Global	1998–2017	PMG ARDL and PDOLS, and GMM	Internet users and Mobile cellular	Negative
Owusu-Agyei et al. [28]	Sub-Saharan Africa	2000–2016	FGLS and 2SLS	Internet users	Positive
Alshubiri et al. [29]	Gulf countries	2000–2016	GMM	Internet users, Mobile cellular, Fixed broadband	Positive
Chien et al. [1]	Global	1990–2015	GMM& PSTR	Fixed broadband	Mixed
Vincent & Evans [30]	Emerging markets	2007–2019	FMOLS	Internet users and Mobile cellular	Positive
Mignamissi & Djijo [26]	Africa	2005–2019	2SLS &GMM	Internet users, Mobile cellular, Fixed broadband	Negative
Ekinci [31]	Turkey	2010–2016	Data Envelopment Analysis	ICT	Positive
Ejemeyovwi et al. [25]	Africa	2000–2017	Bayesian Vector Auto-Regressive	ICT	Positive
Akinlo [32]	Africa	2000–2017	GMM	Internet penetrations, Mobile cellular, Fixed broadband	Mixed
Stakić et al. [33]	Western Balkan Countries	2005–2018	Pooled OLS	Internet users, Mobile cellular	Positive
Pradhan et al. [15]	Asian countries	1991–2012	VAR methods to detect Granger causality	ICT infrastructure	Mixed
Kumar [34]	Vietnam	1980–2018	ARDL	ICT	Positive
Zagorchev et al. [35]	Global	1991–2006	DOLS	Internet users, Mobile cellular,	Positive
Asongu [36]	Africa	2003–2009	OLS	Mobile cellular	Negative
Grahyaia et al. [37]	Saudi Arabia	1990–2019	ARDL	ICT	Positive
Shen et al. [38]	China	2017	Partial Least Squares approach	Internet users,	Positive
Del Gaudio et al. [45]	EU	1995–2015	Fixed effects	ICT	Positive
Mohanty & Bhanumurthy [39]	India	1980–2016	ARDL	ICT infrastructure	Positive

This research is inspired by the contemporary uneven levels of financial development across different income groups and the need to fill a research gap in the existing literature by suggesting a way to enhance financial development across heterogeneous income groups. This study explores the effect of ICT diffusion on financial development for a sample of 37 middle & low-income groups and 42 high-income group economies from 2001 to 2019. The following are the main additions that this article makes to the contemporary research about the connection between ICT and FD. First, unlike most of the previously mentioned research, this research uses a larger data sample encompassing 42 nations, including both high-income and middle and low-income economies. Thus, one of the contributions is to examine disparities between several income-level nations or geographical regions because much of the aforementioned work focuses on underdeveloped nations. Second, Arellano and Bond’s GMM is used in this study to estimate the linear impacts of ICT on FD. Consequently, possible endogeneity, heteroscedasticity, and autocorrelation issues in the data are avoided. In addition, some other techniques, such as fixed effect, random effect, and 2SLS, are employed, allowing us to compare the results of all these techniques with the GMM methods to have more reliable and conclusive estimates., which were missing in the earlier studies. Third, this study provides a comparative analysis that can provide suitable policy implications for different groups of countries. This study’s findings support academicians, financial experts, financial institutions, central banks, commercial banks, foreign investors, the telecommunication industry, and international organizations.

### Theoretical framework, model, and method

Academics have paid a lot of attention to the proliferation of ICT and how it affects the financial sector [40, 41]. Despite the widespread belief that technological advancements have boosted economic development, it is unclear how ICT will affect the banking sector. Solow’s paradox may be used as an initial basis for the topic. Solow describes it well that "you can see the computer age everywhere but in the productivity statistics" [42]. On the one hand, this ambiguity about technical advancements is mostly due to the lack of proof that ICT has a good impact on boosting competitiveness among financial intermediaries [43]. Del Gaudio et al. [44] contend that variations in the econometric approaches used in the literature—more particularly, a lack of agreement on which proxy may most effectively represent the phenomena of technological change—explain the inadequacy of the empirical findings. There is very little literature on the relationship between technical advancements and banking risk, while current studies tend to concentrate on industrial productivity development or examine the consequences of the implementation of a specific technology. Technology enables financial institutions to gather and categorize borrowers more effectively, according to Berger and Mester [45]. Therefore, more information sharing may reduce the likelihood of banking failure and lessen instability. However, Beck et al. [46] demonstrate that financial innovation has a negative impact on the banking sector in terms of both performance and risk.

Theoretically, ICT diffusion affects financial development through four channels: reduced costs, data application, stronger supervision, and positive diffusion. Firstly, the implementation of cloud computing reduces the cost of payment services that only be handled with advanced computing techniques [3]. Secondly, data applications save a lot of time and money by collecting and estimating a large amount of data. Thirdly, ICT become an important tool in the banking sector that enhances their risk management capabilities and helps them correctly estimate loan defaults to safeguard the consumers and protection from money laundering [5]. Moreover, the introduction of blockchain technology in the banking sector protects the data against any hack, manipulation, and theft, thus increasing the privacy of consumers and mutual trust between the concerned parties [47]. Lastly, ICT improves the efficiency of banks and other financial institutions and reduces the information asymmetry in financial transactions, which will benefit the development of the financial sector. On the basis of the above arguments, we develop the following hypothesis: *ICT diffusion promotes financial development*.

As the inclusion of ICT has improved the efficiency of financial markets and reduced the costs of capital, this relationship has grabbed the attention of many empirics and theorists. For instance, Allen et al. [10] emphasize the role of e-financing in the financial sector for reducing information asymmetry between consumers and financial institutions alongside data handling costs. Shamim [13] indicated that FinTech can cut the operational and informational costs that exert a positive impact on economic growth. Moreover, little evidence of the combined effects of financial development and ICT on economic development is also available [48]. Pradhan [15] examines the nexus between ICT, financial development, and economic growth in Asian economies, and their findings confirm the causal link between ICT and financial development. Therefore, following the literature, we have constructed the following model to examine the linkage between ICT and financial development.

$${FD}_{it}=\varphi_{0}+\varphi_{1}{ICT}_{it}+\varphi_{2}{GDP}_{it}+\varphi_{3}{Investment}_{it}+\varphi_{4}{GE}_{it}+\varphi_{5}{Trade}_{it}+\varphi_{6}{IQ}_{it}+\alpha_{i}+\varepsilon_{it}$$

Where financial development (FD) is dependent on information and communication technology (ICT), national income (GDP), Investment, government expenditures (GE), trade openness (Trade), Institutional quality (IQ), country fixed effects (α_i_), and random error term (ε_it_). The subscript i represents the country and t represents the time period. Since prior standard literature [49] confirmed that the level of financial development in the current year is strongly affected by the level of financial development in the previous year, the extended model is written as:

$${FD}_{it}=\varphi_{0}+\lambda_{1}{FD}_{it-1}+\varphi_{1}{ICT}_{it}+\varphi_{2}{GDP}_{it}+\varphi_{3}{Investment}_{it}+\varphi_{4}{GE}_{it}+\varphi_{5}{Trade}_{it}+\varphi_{6}{IQ}_{it}+\alpha_{i}+\varepsilon_{it}$$

As the construction of our equation confirms that our data is the panel, we have shifted our attention toward panel data estimation techniques. When the number of periods is not many or when the time series is not long enough, we don’t employ the panel cointegration techniques rather, we prefer first-generation panel estimation techniques such as "fixed effect (FE), random effect (RE), ordinary least squares (OLS), and generalized methods of moments (GMM)" [50]. There are benefits and drawbacks to both the FE and RE techniques [51]. While RE should be utilized if the cross-sections were generated randomly, FE is appropriate if the cross-sections were selected with confidence. Similarly, the FE approach cannot consider constant factors since this would result in incorrect results. However, since the RE relies on the independence of the regressors and fixed effects, we can include an analysis that includes country-fixed constant factors. These techniques have certain advantages but also some significant drawbacks. For instance, these techniques don’t provide reliable outcomes when "heteroskedasticity, endogeneity, and serial correlation" are present [52]. On the other side, OLS techniques don’t take into consideration the country-specific effects or heterogeneity and time trends. Thus, we have applied the GMM technique proposed by Arellano and Bond [53] to handle all these issues and best suited to dynamic panel data. This technique has various advantages over other methods, such as dealing with the problems of endogeneity, heteroscedasticity, and autocorrelation, among others. Moreover, it delivers a more accommodative variance-covariance setup under moment settings. Undoubtedly, the GMM surpasses the traditional techniques, especially when estimating financial variables, due to its ability to incorporate numerous standard estimators that are simpler than other alternatives [54]. Additionally, this technique is more robust because it works well even if the error terms distribution is incorrect. Last but not least, the Sargan test allows us to examine the over-acknowledgment of instrument variables and to check whether the tool variables are independent of error terms or not.

### Data and descriptive analysis

This study explores the effect of ICT diffusion on financial development for a sample of 37 middle & low-income groups and 42 high-income group economies for the period 2001 to 2019.Table A1 in S1 Appendix provides the list of the sample of the economies employed in this study. Following the standard literature [26], three measures of financial sector development are used. The first variable shows the overall performance of financial development. The other two variables represent the performance of financial institutions and financial markets, respectively. We purposely included the financial institution’s development and financial markets development for the current study. Nguyen et al. [22] argued that the contribution of financial institutions and markets is more important in the digital economy. Data for the financial development index, financial markets index, and financial institutions index have been obtained from the IMF. Similarly, several measures of ICT diffusion have been used in the literature. For instance, Cheng et al. [55] use internet users, mobile cellular, and secure internet servers as a measure of ICT diffusion. Most recently, Chien et al. [1] suggested internet users, mobile cellular, and fixed telephone as a proxy for capturing the impact of ICT diffusion. Based on the literature, we have constructed ICT variables by using three different ICT-related variables such as internet users, mobile cellular, and fixed broadband. Nguyen et al. [22] reasoned that the economic performance of any country and its connectivity with the global world via ICT has important implications on the performance of financial sector development. Data for mobile cellular, internet users, and fixed broadband subscriptions have been extracted from the World Bank.

This study has also generated an index for ICT diffusion by utilizing the principal component analysis (PCA) approach. Following Pradhan et al. [15], the study developed an ICT diffusion index from the variables internet, mobile, and fixed broadband. In addition to the indicators of financial development and ICT diffusions, the study employs a set of control variables associated with ICT and financial development. In this regard, economic and institutional factors are used. The study used GDP per capita, government consumption expenditures, investment, trade, and law& order as control variables. Owusu-Agyeiet al. [28] suggested that as the economic activities of the country grow, it tends to increase the financial sector development. Similarly, trade openness [28], government expenditure [29], and investment [22] can affect the financial sector development by increasing capital formation, demand for financial goods, credit availability, economic restructuring, investment, competition, and capital inflows. Data for GDP per capita, government expenditure, trade openness, and investment are taken from the World Bank. Following the standard practice [56], we used law & order as an indicator of institutional quality, and data for this variable is extracted from the ICRG.

Table 2 displays detailed information regarding descriptive statistics, symbols, definitions of variables, and sources of data. The mean of FD, FID, FMD, ICT, Internet, Mobile, FBS are 0.305, 0.364, 0.237, 36.83, 29.02%, 78.50%, and 4.365% in middle & low-income group, while 0.596, 0.658%, 0.516%, 62.23, 65.56%, 107.8%, and 21.14% in high-income group. This infers that financial development and ICT diffusion is more in high-income countries compared to middle & low-income countries. The economic performance and law and order situation of high-income countries is also better than the middle & low-income countries. Before the execution of regression analysis, we first check the correlation among variables. Table 3 displays the results of the correlation matrix among the concerned variables. Findings indicate that a higher correlation exists among mobile cellular, internet, fixed broadband subscriptions, and ICT. However, this high correlation does not infer the existence of correlation among these variables because mobile cellular, internet, and fixed broadband subscriptions are three proxies of ICT diffusion, which are regressed in separate models.

**Table 2:** Variables and data description.

		Middle & Low-Income group		High-income group		
Variable	Definition	Mean	S.D	Mean	S.D	Sources
FD	Financial Development Index	0.305	0.158	0.596	0.204	IMF
FID	Financial Institutions Index	0.364	0.139	0.658	0.178	IMF
FMD	Financial Markets Index	0.237	0.212	0.516	0.265	IMF
ICT	ICT index	36.83	22.63	62.23	18.59	WDI
Internet	Individuals using the Internet (% of population)	29.02	22.54	65.56	23.18	WDI
Mobile	Mobile cellular subscriptions (per 100 people)	78.50	45.65	107.8	29.82	WDI
FBS	Fixed broadband subscriptions (per 100 people)	4.365	5.507	21.14	12.69	WDI
GDP	GDP per capita (constant 2015 US$)	8.284	0.703	10.23	0.577	WDI
GE	General government final consumption expenditure (% of GDP)	13.48	3.676	18.96	4.022	WDI
Investment	Gross fixed capital formation (% of GDP)	23.62	6.721	22.72	4.194	WDI
Trade	Trade (% of GDP)	71.70	34.71	102.7	63.49	WDI
Lorder	Law and order index ranges from 0 to 6	3.128	0.920	4.982	0.763	ICRG

**Table 3:** Correlations matrix.

**Middle & Low-Income group**													
		1	2	3	4	5	6	7	8	9	10	11	12
1	FD	1											
2	FID	0.821	1										
3	FMD	0.927	0.549	1									
4	Internet	0.333	0.556	0.123	1								
5	Mobile	0.299	0.508	0.105	0.763	1							
6	FBS	0.334	0.504	0.159	0.799	0.588	1						
7	ICT	0.337	0.564	0.124	0.908	0.961	0.741	1					
8	GDP	0.397	0.531	0.234	0.537	0.460	0.525	0.528	1				
9	Consumption	0.239	0.458	0.050	0.216	0.223	0.207	0.237	0.253	1			
10	Investment	0.120	0.047	0.145	0.058	0.083	0.158	0.087	0.089	-0.032	1		
11	Trade	0.171	0.228	0.101	0.160	0.170	0.046	0.170	0.116	0.002	0.158	1	
12	Lorder	0.073	0.012	0.099	0.003	-0.116	-0.003	-0.074	-0.159	0.172	0.314	0.282	1
**High-income group**													
		1	2	3	4	5	6	7	8	9	10	11	12
1	FD	1											
2	FID	0.858	1										
3	FMD	0.939	0.630	1									
4	Internet	0.373	0.383	0.308	1								
5	Mobile	0.013	-0.020	0.033	0.552	1							
6	FBS	0.401	0.431	0.319	0.847	0.512	1						
7	ICT	0.268	0.264	0.229	0.906	0.836	0.857	1					
8	GDP	0.763	0.690	0.693	0.508	0.111	0.474	0.392	1				
9	Consumption	0.038	0.020	0.045	0.171	0.066	0.179	0.150	0.078	1			
10	Investment	0.020	0.006	0.026	-0.019	-0.020	-0.140	-0.051	0.042	-0.244	1		
11	Trade	-0.199	-0.178	-0.182	0.061	0.197	0.083	0.139	-0.041	-0.234	0.149	1	
12	Lorder	0.533	0.448	0.507	0.351	-0.046	0.241	0.192	0.646	0.368	0.084	-0.005	1

## Results and discussion

Table 4 displays the findings of ICT diffusion on financial development for both samples. Models 1 to 4 report the coefficient estimates for the low & middle-income groups while models 5 to 8 report the coefficient estimates for high-income group economies. It is found that the coefficient of the internet is statistically insignificant in all the models demonstrating that the internet does not contribute to financial development in low, middle, and high-income economies. The coefficient of mobile is negative and significant in the middle & low-income groups while it is positive and significant in the high-income group, implying that the use of mobile discourages financial development in the middle & low-income groups, but encourages financial development in the high-income group. The coefficient of mobile is -0.006 in the middle & low-income group and 0.019 in the high-income group. Fixed broadband subscription impact is insignificant in low & middle-income groups, in contrast, the impact of fixed broadband subscription is found significant and positive in high-income group economies with a coefficient estimate of 0.008. The ICT index is found negative and significant in the middle & low-income group with a coefficient estimate of -0.006, while it is positive and significant in the high-income group with a coefficient estimate of 0.018. All categories of ICT diffusion, except the internet, report a positive impact on financial development in high-income groups revealing that higher financial development in these economies comes from ICT diffusion. Our empirical evidence validated the hypothesis within the high-income group, whereas it was rejected within the middle and low-income groups.

**Table 4:** ICT diffusion and financial development.

	Middle & Low-Income group				High-income group			
	(1)	(2)	(3)	(4	(5)	(6)	(7)	(8)
L.FD	0.798***	0.802***	0.797***	0.799***	0.692***	0.674***	0.685***	0.675***
	(0.027)	(0.025)	(0.029)	(0.025)	(0.027)	(0.030)	(0.030)	(0.028)
Internet	-0.001				0.008			
	(0.003)				(0.006)			
Mobile		-0.006**				0.019***		
		(0.003)				(0.007)		
FBS			0.001				0.008***	
			(0.001)				(0.001)	
ICT				-0.006**				0.018***
				(0.003)				(0.007)
GDP	0.011	0.020**	0.010	0.019**	0.031**	0.020	0.029**	0.026*
	(0.008)	(0.008)	(0.008)	(0.008)	(0.014)	(0.015)	(0.014)	(0.015)
Consumption	0.006	0.007	0.006	0.007	0.023**	0.015	0.022*	0.018
	(0.006)	(0.005)	(0.005)	(0.005)	(0.012)	(0.013)	(0.012)	(0.013)
Investment	0.012**	0.013**	0.012**	0.013**	0.031***	0.025**	0.036***	0.026***
	(0.006)	(0.005)	(0.006)	(0.006)	(0.009)	(0.010)	(0.010)	(0.010)
Trade	0.008	0.011	0.008	0.011	0.005	-0.001	-0.007	-0.001
	(0.009)	(0.007)	(0.008)	(0.008)	(0.013)	(0.012)	(0.014)	(0.013)
Lorder	0.003	0.004	0.003	0.004	-0.001	0.001	0.000	-0.001
	(0.003)	(0.003)	(0.003)	(0.003)	(0.007)	(0.007)	(0.006)	(0.007)
Constant	-1.082	-1.475**	-0.482	-1.686***	1.784*	1.954***	3.606***	2.483***
	(1.109)	(0.578)	(0.586)	(0.626)	(0.931)	(0.591)	(1.057)	(0.809)
Observations	629	629	629	629	714	714	714	714
Number of code	37	37	37	37	42	42	42	42
Sargan Test	0.424	0.495	0.439	0.474	0.198	0.162	0.203	0.137

Notes

a. *** p<0.01, ** p<0.05, * p<0.1

b. The number of observations in the middle & low-income economies is 629, while in high income economies are 714.

Findings of middle & low-income groups are also consistent with Nguyen et al. [22] who inferred that ICT diffusion impact is negative on financial development in middle & low-income groups due to insufficient technological innovation and lack of communication infrastructures. Another fact is that in the period of post-crisis, fixed broadband subscriptions and mobile may have disseminated negative news that resulted in harming financial development and halted the development of financial markets due to insufficient capacity for checking information. ICT diffusion has a negative impact on financial development by economic growth channels [56]. Regarding high-income groups, our findings suggest that the ICT diffusion contributes to the development of the financial sector in high-income. ICTs include mobile phones, the internet, and other information technologies that help to dematerialize the economy and enable every sector in the economy to work more efficiently and at a reduced cost in high-income. Banks and other financial institutions can use the internet and mobile services to disseminate information about their products. On the demand side, the increased use of the internet and mobile phones allows people to gather information about financial products and services without any hassle. People can now make online accounts by using mobile applications and can do transactions worth millions through mobiles and laptops which all contribute to the financial development and economic growth of a nation.

Regarding the direct impact of ICT diffusion on financial development, improved ICT diffusion may cause the “knowledge asymmetry” between lenders and borrowers to fall. In fact, banks may rate their borrowers more favorably thanks to increased information availability [57]. Therefore, more information sharing might reduce the likelihood of bank failure and increase stability. In reality, ICT dissemination may lead to a decline in physical branches, their related expenses, and their replacement. According to Aminizadeh et al. [58], internet delivery routes may provide advantageous economies of scale that may contend with conventional distribution methods. The progress of ICT can halt the growth of the traditional financial sector because ICT diffusion encourages the development of financial technology (FinTech), which is the primary reason behind the transformation of financial institutions. With the introduction of FinTech, banks, and other financial institutions can provide the same services in a much more efficient and cost-effective way, relying on a distinct and unbundled mode [59]. Mobile phones and the internet have become crucial for financial development because they effectively reduce the cost of the services provided by financial intermediaries such as commercial and microfinance banks [44]. Moreover, ICTs help improve the process of information collection, which allows financial institutions to scrutinize applicant creditworthiness more efficiently to expedite deposits.

The positive effects of ICT diffusion on financial development are also supported by some past studies. For instance, Prucell and Toland [12] postulate that ICT can help in developing and sustaining credit information databases that will help banking and other financial institutions to work more efficiently. Asongu et al. [17] highlighted that ICT diffusion successfully counters the adverse effects of market authority on the quantity and cost of credits. They also pointed out that the market power can be overcome with the moderating effect of ICT, thus improving access to financial products and services. In another study on African economies, Asongu [20] considered ICT among the four variables of the knowledge economy index and observed that it improves the efficiency of all financial sectors. The positive impact of ICT is also supported by Gupta & Kanungo [23], who noted that ICT allows people in remote or underserved areas to access financial services. Mobile banking enables individuals to open accounts, make payments, and access credit without the need for a physical bank branch. This also means that ICT reduces the cost of providing financial services. It also streamlines financial processes.

In terms of control variables, the impact of GDP is found significant and positive on financial development in two models of the middle & low-income group and three models of the high-income group. The impact of consumption is significant and positive on financial development in two models of the high-income group only. Investment variable impact is significant and positive on financial development in all eight models; in contrast, trade and law & order produce no impact on financial development in any model.

Table 5 displays the findings of ICT diffusion on financial markets development for middle & low-income and high-income groups. The results indicate that the internet role is again insignificant in defining the performance of financial markets development in both samples. The findings display that mobile has a significant and negative impact on financial markets development in middle & low-income countries, while it shows a significant and positive impact on financial markets development in high-income groups. An upsurge in mobile increases financial markets development by 0.018% in high-income groups and reduces financial markets development by 0.010% in middle & low-income groups. It is further reported that fixed broadband subscriptions impact is insignificant in the case of middle & low-income groups; however, fixed broadband subscription produces a significant and positive impact on financial markets development with a coefficient estimate of 0.003%. Finally, an increase in ICT diffusion tends to discourage financial markets development by 0.010% in middle & low-income groups. However, the impact of ICT diffusion is statistically insignificant on financial markets development in high-income groups. It is reported that the impact of GDP and consumption is insignificant on financial markets development in both samples. However, investment impact is significant and positive on financial markets development in two models of middle & low-income groups and all four models of the high-income group. In the end, trade impact on financial markets development is observed significant and positive in three models of the high-income group only. Law & order report a significant and positive impact on financial markets development in all four models of middle & low-income groups only.

**Table 5:** ICT diffusion and financial markets development.

	Middle & Low-Income group				High-income group			
	(1)	(2)	(3)	(4	(5)	(6)	(7)	(8)
L.FMD	0.774***	0.779***	0.774***	0.779***	0.646***	0.636***	0.643***	0.641***
	(0.039)	(0.036)	(0.043)	(0.036)	(0.036)	(0.035)	(0.036)	(0.036)
Internet	-0.003				0.001			
	(0.005)				(0.007)			
Mobile		-0.010**				0.018*		
		(0.004)				(0.011)		
FBS			0.001				0.003***	
			(0.001)				(0.001)	
ICT				-0.010***				0.010
				(0.004)				(0.009)
GDP	0.002	0.023	-0.001	0.021	0.010	0.000	0.008	0.008
	(0.016)	(0.015)	(0.015)	(0.015)	(0.015)	(0.0190	(0.017)	(0.017)
Consumption	0.003	0.005	0.002	0.005	-0.004	-0.015	-0.011	-0.011
	(0.010)	(0.010)	(0.009)	(0.010)	(0.023)	(0.0250	(0.023)	(0.024)
Investment	0.013	0.015**	0.014	0.015**	0.045***	0.039**	0.050***	0.040***
	(0.008)	(0.007)	(0.009)	(0.008)	(0.016)	(0.016)	(0.016)	(0.016)
Trade	0.015	0.020	0.013	0.020	0.047*	0.042*	0.036	0.045*
	(0.016)	(0.014)	(0.014)	(0.014)	(0.025)	(0.024)	(0.026)	(0.025)
Lorder	0.009*	0.011**	0.008*	0.011**	0.001	0.001	-0.002	-0.001
	(0.005)	(0.005)	(0.004)	(0.005)	(0.013)	(0.013)	(0.013)	(0.013)
Constant	-0.329	-1.206	0.951	-1.549	0.729	1.458	4.819***	1.596
	(1.899)	(1.143)	(1.125)	(1.212)	(1.533)	(1.278)	(1.738)	(1.441)
Observations	629	629	629	629	714	714	714	714
Number of code	37	37	37	37	42	42	42	42
Sargan Test	0.225	0.312	0.211	0.170	0.162	0.190	0.285	0.654

Notes

*** p<0.01, ** p<0.05, * p<0.1

The number of observations in the middle & low-income economies is 629, while in high-income economies is 714.

Table 6 reports the results of ICT diffusion on financial institutions development for middle & low-income and high-income groups. It is observed that ICT diffusion does not produce any impact on financial institutions’ development in middle & low-income groups as the coefficient estimates for internet, mobile, FBS, and index of ICT are statistically insignificant. However, internet, mobile, and ICT show a significant and positive impact on financial institutions’ development for the sample of the high-income group. It is reported that an increase in usage of the internet and mobile increases financial institutions’ development by 0.011% and 0.018% percent respectively. Moreover, a 0.018% increase in financial institutions’ development is observed due to a unit increase in the ICT index. Findings infer that GDP produces a significant and positive impact on financial institutions’ development in all four models of middle & low-income groups only. Consumption has a significant and positive effect on financial institutions’ development in two models of the middle & low-income group and all four models of the high-income group. Investment reports a positive and significant impact on financial institutions’ development in all the models of both samples. However, trade reports a significant and negative impact on financial institutions’ development in all four models of high-income groups. Law & order has an insignificant impact on financial institutions’ development in all eight models.

**Table 6:** ICT diffusion and financial institutions development.

	Middle & Low-Income group				High-income group			
	(1)	(2)	(3)	(4	(5)	(6)	(7)	(8)
L.FID	0.842***	0.848***	0.849***	0.846***	0.806***	0.796***	0.819***	0.794***
	(0.030)	(0.030)	(0.034)	(0.031)	(0.028)	(0.026)	(0.029)	(0.027)
Internet	0.001				0.011**			
	(0.003)				(0.005)			
Mobile		0.001				0.018***		
		(0.002)				(0.004)		
FBS			0.001				0.001	
			(0.001)				(0.001)	
ICT				0.001				0.018***
				(0.003)				(0.004)
GDP	0.018***	0.016**	0.020***	0.015**	0.023	0.012	0.018	0.019
	(0.007)	(0.008)	(0.007)	(0.008)	(0.018)	(0.016)	(0.016)	(0.017)
Consumption	0.008*	0.008	0.007*	0.008	0.034**	0.026*	0.035**	0.029*
	(0.005)	(0.005)	(0.004)	(0.005)	(0.017)	(0.014)	(0.016)	(0.016)
Investment	0.012*	0.011*	0.012**	0.011*	0.024***	0.017**	0.027***	0.019**
	(0.007)	(0.006)	(0.006)	(0.006)	(0.009)	(0.009)	(0.008)	(0.009)
Trade	0.001	0.001	0.002	0.001	-0.027**	-0.033***	-0.029***	-0.033***
	(0.006)	(0.006)	(0.006)	(0.007)	(0.011)	(0.011)	(0.011)	(0.012)
Lorder	-0.003	-0.002	-0.002	-0.002	0.000	0.002	0.002	0.001
	(0.002)	(0.002)	(0.003)	(0.002)	(0.005)	(0.004)	(0.004)	(0.004)
Constant	-0.970	-0.922	-1.177**	-0.922	2.202***	1.944***	1.055	2.583***
	(1.087)	(0.772)	(0.558)	(0.911)	(0.647)	(0.5370	(0.779)	(0.601)
Observations	629	629	629	629	714	714	714	714
Number of code	37	37	37	37	42	42	42	42
Sargan Test	0.594	0.752	0.179	0.282	0.782	0.842	0.717	0.775

Notes: Robust standard errors in parentheses

*** p<0.01

** p<0.05

* p<0.1

## Conclusion and policy implications

Over the past few years, the growth of ICTs, such as mobile phones and the Internet, has revolutionized the social and economic structure of the nations. Recent advancements in ICT have benefited every sector of the economy, and the financial industry is no exception. ICT has completely altered the methods of processing information used by financial institutions. ICT has become increasingly important for the global financial industry because it allows financial institutions and markets to work more efficiently. Hence, the investment of the financial sector in ICT soared to US$197 billion in 2014, which is the highest investment in the ICT sector by any industry since 1995 [1]. ICT diffusion has also redefined the pace and type of financial development. For example, existing business firms can take advantage of the new commercial options through alteration in the methods of product value creation and delivery services presented by ICT innovations in the business model. Given the importance of ICT diffusion in the development of the financial sector, empirics have turned their attention to this relationship. Following the recent trends, this analysis is an effort to analyze the transmission channels between ICT diffusion and financial sector’s development in high-income economies as well as in middle and low-income economies. To make our results reliable and robust, we have used different variables to represent ICT and financial development. We employed the GMM method in our empirical analysis.

Our results indicate that the estimates of mobile cellular subscription and ICT index in the financial development model are negative in middle and low-income economies and positive in high-income economies. The estimates of fixed broadband subscriptions and internet users are insignificant in middle and low-income economies. However, the estimate of fixed broadband subscriptions is positive in high-income economies; whereas, the estimate of internet users is insignificant. Similarly, the estimated coefficients of mobile subscribers are positively significant in the financial market model in both income groups. On the other side, the estimate of ICT is negatively significant only in the middle and low-income economies, and the estimate of fixed broadband subscribers is only significant in the high-income group. Lastly, in the financial institution model, the estimates of internet users, mobile subscribers, and ICT index are positively significant in the high-income group and insignificant in the middle and low-income. We can conclude our results by saying that ICT diffusion positively impacts financial development in high-income economies and negatively impacts middle and low-income economies. Further, among different measures of ICT, mobile cellular subscription turns out to be most crucial for financial development in both income groups, though the effects are opposite in both income groups.

The results are crucial for policy discussion in both income groups. First, we discuss the policy implications for middle and low-income economies and then for high-income economies.

### Middle and low-income economies

Middle- and low-income-economy policymakers should follow the footprint of the high-income economies and increase the role of ICT in the financial sector for its development. First, middle- and low-income governments should prioritize investments in ICT infrastructure, especially broadband connections and mobile networks,to enable universal access to digital financial services. Financial inclusion may be improved by extending digital infrastructure to underserved rural communities. Second, to promote innovation, middle- and low-income economic policymakers should foster an environment that prioritizes consumer safety and financial stability. This involves resolving problems with cybersecurity, data privacy, and digital identity. Lastly, policymakers in low- and middle-income countries should encourage the use of digital payment methods, such as mobile money and digital wallets, to speed up financial inclusion. Governments may promote the adoption of digital payments for both private citizens and commercial enterprises, lowering the dependency on cash transactions.

### High-income economies

Since the financial sector in high-income economies is already equipped with ICT and related applications and tools, the policymakers in these economies should take more and more benefits from using ICT in developing their financial sectors. In this regard, we suggest the following to the policymakers in high-income economies. First, policymakers in high-income economies should make a regulatory sandbox focused on digital innovation unique to fintech developments. The review and approval of digital financial services and technology would be given priority in this sandbox, enabling them to reach the market more quickly while maintaining regulatory compliance and consumer safety. Second, policymakers should examine how machine learning and artificial intelligence (AI) may be used to improve regulatory skills. With these technologies, authorities may more effectively monitor market activity in real-time, identify developing dangers, and determine compliance with intricate laws. Third, policymakers in high-income economies should promote the use of blockchain and smart contracts in financial institutions. In fields including trade finance, supply chain financing, and digital identity verification, these technologies may improve procedures, reduce fraud, and increase transparency. Fourth, policymakers should encourage financial firms to embrace regulatory technology (regtech) solutions. Give institutions incentives to use regtech technologies that automate compliance activities, lowering regulatory burdens and increasing efficiency. Lastly, policymakers should promote digital inclusion by ensuring all residents can access fundamental financial services. To guarantee that no one is left behind in the financial digital revolution, implement policies that address the digital gap, especially among marginalized communities.

Even though this research sheds light on how ICT spread and financial development relate across a range of nations, it nonetheless has several important limitations. First and foremost, the analysis has only divided the selected economies based on their income levels and ignored various other factors on which countries can be collected, such as economic blocs (BRICS, European Union, etc.), trade blocs (NAFTA, SAFTA, etc.), and regional blocs (SAARC, MENA, ASEAN, etc.). Thus, to add more value to future analysis, the empirics should focus on analyzing the relationship in the context of regional, trade, and economic blocs. Another important limitation of the analysis is that it ignores the asymmetric impacts that are present in most macro variables. Thus, future empirics can focus on analyzing the nonlinear effects of ICT diffusion on financial development. Lastly, cross-sectional dependence is an important issue in a panel data analysis for which our methodology doesn’t account; thus, future analysis should estimate the impact of ICT diffusion on financial development with the advanced panel data techniques that can account for cross-sectional dependence (S1 Dataset).

## Supporting information

S1 Dataset (XLSX)

S1 Appendix (DOCX)
