# Supplementary material for: ICT diffusion and financial development: Comparing high, middle, and low-income countries
Source: PLoS One. 2024 May 2;19(5):e0295183. doi: 10.1371/journal.pone.0295183 (PMC11065209; doi:10.1371/journal.pone.0295183)
Supplement: S1 Appendix — (DOCX) [file pone.0295183.s002.docx]

**Appendix**

**Table A1: List of Middle & Low-income and High-income Countries**

| **High-Income countries** | | | | **Middle & Low-Income countries** | | | |
| --- | --- | --- | --- | --- | --- | --- | --- |
| **No** | **Country** | **No** | **Country** | **No** | **Country** | **No** | **Country** |
| 1 | Australia | 22 | Japan | 1 | Algeria | 22 | Moldova |
| 2 | Austria | 23 | Korea, Rep. | 2 | Armenia | 23 | Morocco |
| 3 | Bahamas | 24 | Latvia | 3 | Azerbaijan | 24 | Nigeria |
| 4 | Bahrain | 25 | Lithuania | 4 | Bolivia | 25 | Pakistan |
| 5 | Belgium | 26 | Malta | 5 | Brazil | 26 | Panama |
| 6 | Canada | 27 | Netherlands | 6 | Burkina Faso | 27 | Paraguay |
| 7 | Chile | 28 | New Zealand | 7 | China | 28 | Peru |
| 8 | Croatia | 29 | Norway | 8 | Colombia | 29 | Philippines |
| 9 | Cyprus | 30 | Oman | 9 | Costa Rica | 30 | Romania |
| 10 | Czech Republic | 31 | Poland | 10 | Dominican Republic | 31 | Russian Federation |
| 11 | Denmark | 32 | Portugal | 11 | Ecuador | 32 | Senegal |
| 12 | Estonia | 33 | Saudi Arabia | 12 | Egypt, Arab Rep. | 33 | South Africa |
| 13 | Finland | 34 | Singapore | 13 | El Salvador | 34 | Thailand |
| 14 | France | 35 | Slovak Republic | 14 | Gabon | 35 | Tunisia |
| 15 | Germany | 36 | Slovenia | 15 | India | 36 | Turkey |
| 16 | Greece | 37 | Spain | 16 | Indonesia | 37 | Vietnam |
| 17 | Hungary | 38 | Sweden | 17 | Iran |  |  |
| 18 | Iceland | 39 | Switzerland | 18 | Jamaica |  |  |
| 19 | Ireland | 40 | United Kingdom | 19 | Jordan |  |  |
| 20 | Israel | 41 | United States | 20 | Malaysia |  |  |
| 21 | Italy | 42 | Uruguay | 21 | Mexico |  |  |
